# Phenotype and specificity of T cells in primary human cytomegalovirus infection during pregnancy: IL-7R^pos^ long-term memory phenotype is associated with protection from vertical transmission

**DOI:** 10.1371/journal.pone.0187731

**Published:** 2017-11-07

**Authors:** Federico Mele, Chiara Fornara, David Jarrossay, Milena Furione, Alessia Arossa, Arsenio Spinillo, Antonio Lanzavecchia, Giuseppe Gerna, Federica Sallusto, Daniele Lilleri

**Affiliations:** 1 Center of Medical Immunology, Institute for Research in Biomedicine, Università della Svizzera Italiana, Bellinzona, Switzerland; 2 Laboratori Sperimentali di Ricerca-Area Trapiantologica and Area Biotecnologie, Fondazione IRCCS Policlinico San Matteo, Pavia, Italy; 3 Immune Regulation Laboratory, Institute for Research in Biomedicine, Università della Svizzera Italiana, Bellinzona, Switzerland; 4 Struttura Semplice Virologia Molecolare, Struttura Complessa Microbiologia e Virologia, Fondazione IRCCS Policlinico San Matteo, Pavia, Italy; 5 Clinica Ostetrica e Ginecologica, Universita’ di Pavia, Fondazione IRCCS Policlinico San Matteo, Pavia, Italy; 6 Institute of Microbiology, ETH Zurich, Zurich, Switzerland; University of Arizona, UNITED STATES

## Abstract

Congenital human cytomegalovirus (HCMV) infection is the major cause of birth defects and a precise definition of the HCMV-specific T-cell response in primary infection may help define reliable correlates of immune protection during pregnancy. In this study, a high throughput method was used to define the frequency of CD4^+^ and CD8^+^ T cells specific for four HCMV proteins in the naïve compartment of seronegative subjects and the effector/memory compartments of subjects with primary/remote HCMV infection. The naïve repertoire displayed comparable frequencies of T cells that were reactive with HCMV structural (pp65, gB and the pentamer gHgLpUL128L) and non-structural (IE-1) proteins. Whereas, following natural infection, the majority of effector/memory CD4^+^ and CD8^+^ T cells recognized either gB or IE-1, respectively, and pp65. The pattern of T cell reactivity was comparable at early and late stages of infection and in pregnant women with primary HCMV infection transmitting or not transmitting the virus to the fetus. At an early stage of primary infection, about 50% of HCMV-reactive CD4^+^ T cells were long-term IL-7R^pos^ memory cells, while 6–12 months later, the frequency of these cells increased to 70%, approaching 100% in remote infections. In contrast, only 10–20% of HCMV-specific CD8^+^ T cells were long-term memory cells up to 12 months after infection onset, thereafter increasing to 70% in remote infections. Interestingly, a significantly higher frequency of HCMV-specific CD4^+^ T cells with a long-term IL-7R^pos^ memory phenotype was observed in non-transmitting compared to transmitting women. These findings indicate that immunodominance in HCMV infection is not predetermined in the naïve compartment, but is the result of virus-host interactions and suggest that prompt control of HCMV infection in pregnancy is associated with the rapid development of long-term IL-7R^pos^ memory HCMV-specific CD4^+^ T cells and a low risk of virus transmission to the fetus.

## Introduction

Human cytomegalovirus (HCMV) is the most common cause of congenital infection, and may lead to mental retardation, psychomotor delay, hearing loss, speech and language disabilities, behavioral disorders and visual impairment. Vertical transmission occurs in about 0.6% of pregnancies [1], and the infected fetus may present with symptoms at birth or develop severe long-term *sequelae* (in about 20% of cases) [2, 3]. Although both primary and non-primary infections during pregnancy may cause congenital infections, severe symptoms at birth and long-term *sequelae* are more commonly observed in infected infants born to mothers experiencing HCMV primary infection during pregnancy [4], when about 40% fetuses develop HCMV infection *in utero* [5, 6]. To date, no viral or host factor has been definitively associated with HCMV transmission to the fetus.

In previous studies, we provided evidence that delayed T and B cell responses to HCMV primary infection in pregnancy are associated with virus transmission to the fetus [7–12]. In this study, we extended the analysis of the development of T-cell responses to HCMV and their relationship with congenital HCMV infection after primary infection in pregnancy. We used a high throughput cell-based screening assay [13] to measure, with high sensitivity, the frequencies of HCMV-specific T cells in naïve and effector/memory subsets of HCMV seronegative and seropositive donors and patients following primary HCMV infection, including pregnant women transmitting (T) or non-transmitting (NT) the virus to the fetus.

The method adopted is based on the screening of T-cell libraries grown under culture conditions that allow even expansion of polyclonal T cells [13]. With respect to other direct *ex vivo* methods for detecting antigen specific T cells (such as cytokine production or activation marker expression), this method has sufficient sensitivity to detect antigen-specific T cells when their frequency is low (as occurs in the naïve repertoire and in memory T cells specific for poorly represented antigens) and allows analysis of multiple antigen specificities even when the available sample is small.

Out of the 150 HCMV open reading frames previously found to elicit a CD4^+^ or CD8^+^ T-cell response [14], we selected four broadly recognized HCMV proteins, which have been widely investigated in vaccine and immune monitoring studies and are representative of different synthesis kinetics and virion structures: the non-structural IE-1 protein (produced in the immediate-early phase, before virus DNA replication), the structural proteins pp65 (internal tegument protein, produced in excess during virus replication), and the envelope glycoprotein complexes including the pentamer gHgLpUL128-130-131 (gHgLpUL128L) and gB.

We analysed the distribution of HCMV-specific lymphocytes among naïve T cells and two subsets of antigen experienced T cells. The latter are characterized by the different expression of interleukin-7 receptor α chain (IL-7R): IL-7R^pos^ “long-term” memory cells and IL-7R^neg^ “short-term” effector cells [15–17].

Interestingly, we found that the rapid development of long-term memory HCMV-specific CD4^+^ T cells in pregnant women with primary infection is associated with a lower risk of HCMV transmission to the fetus.

## Results

### Dissection of HCMV protein T-cell specificity

**Fig 1:**
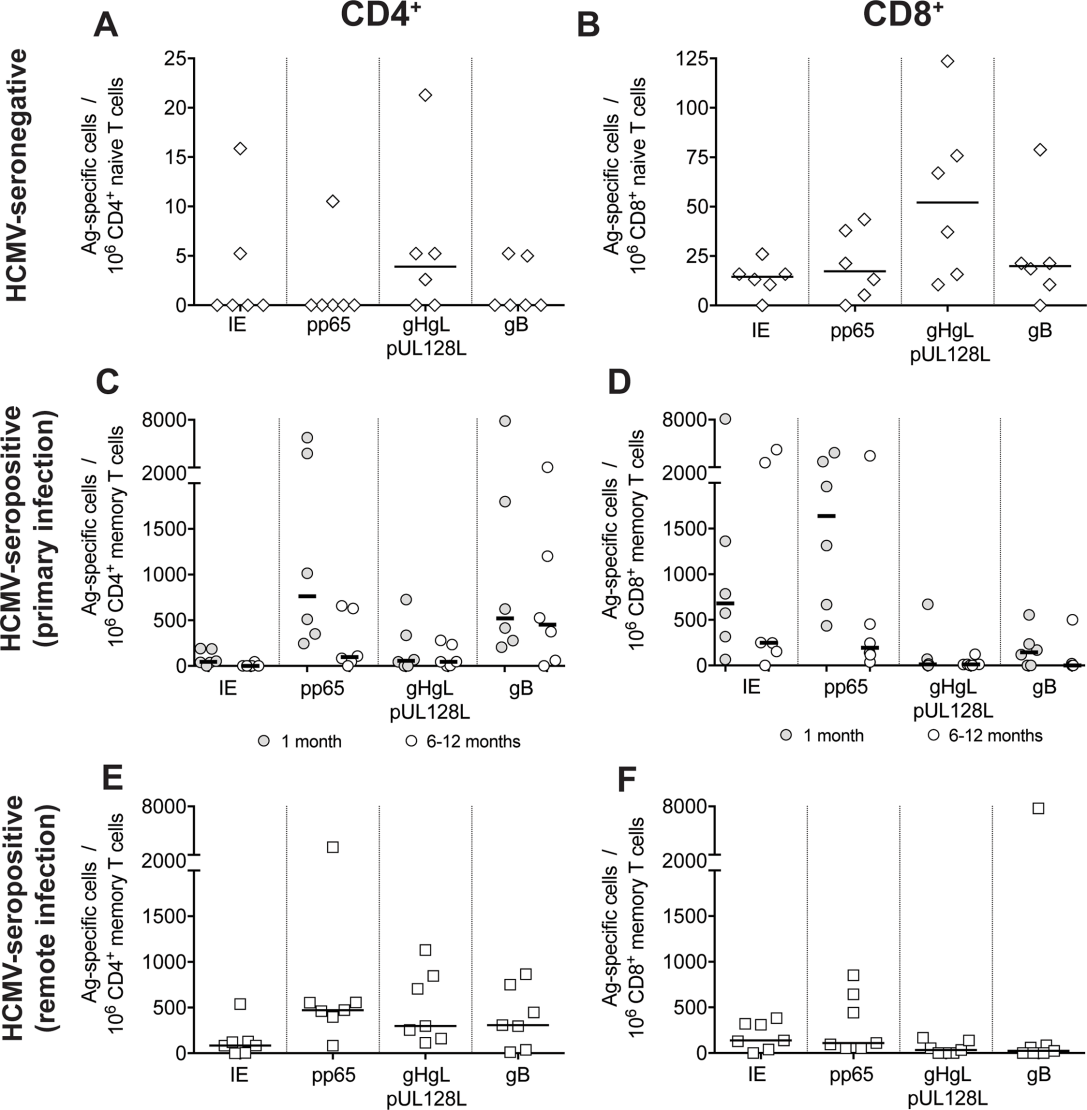
Frequencies of CD4^+^ and CD8^+^ T cells specific for HCMV proteins IE-1, pp65, gHgLpUL128L (pentamer) and gB in the naïve pool of HCMV-seronegative subjects and in the memory pool of subjects with primary or remote HCMV infection. **A, B.** Frequencies of protein-specific **(A)** CD4^+^ and **(B)** CD8^+^ naïve T cells in 6 HCMV-seronegative subjects are reported. Each symbol represents an individual and horizontal black lines indicate median values. **C, D.** Frequencies of protein-specific **(C)** CD4^+^ and **(D)** CD8^+^ memory T cells in 6 patients with primary HCMV infection tested within one month and 6–12 months after infection onset. **E, F.** Frequencies of protein-specific **(E)** CD4^+^ and **(F)** CD8^+^ memory T cells in 7 subjects with remote HCMV infection are reported.

As a first step, we measured the frequencies of HCMV-specific CD4^+^ and CD8^+^ T-cells in the naïve repertoire of six HCMV-seronegative healthy donors, and in the memory compartment of six patients with primary HCMV infection and seven HCMV-seropositive healthy donors with remote (i.e. occurring >5 years before) infection (Fig 1). Patients with primary HCMV infection (Table 1) were tested in the acute (i.e. 1 month after onset) and late (6–12 months after onset) stages of infection.

**Table 1:** Characteristics of the 27 patients with primary HCMV infection.

Parameter	Non-pregnant (n = 6)	Non-transmitter mothers (n = 10)	Transmitter mothers (n = 11)
Age, median (range)	40 (29–73)	35 (29–42)	35 (29–41)
Gestational week at onset of infection, median (range)	Not applicable	20 (6–26)	23 (6–25)
No. symptomatic infections	6/6	8/10	11/11
Sample collection—No. of days after onset of infection, median (range):			
- acute stage	23 (13–26)	22 (13–34)	28 (16–33)
- late stage	268 (176–380)	280 (200–380)	384 (195–437)
No. subjects with CD4^+^ T-cells specific for:			
- IE-1	5/6	9/10	9/11
- pp65	6/6	10/10	11/11
- gHgLpUL128L (pentamer)	6/6	10/10	11/11
- gB	6/6	10/10	11/11
No. subjects with CD8^+^ T-cells specific for:			
- IE-1	6/6	10/10	11/11
- pp65	6/6	9/10	10/11
- gHgLpUL128L (pentamer)	4/6	7/10	9/11
- gB	4/6	9/10	10/11

Naive (CD45RA^+^ CCR7^+^) and memory (comprising both CD45RA^−^CCR7^+^ central memory and CD45RA^–^/CD45RA^+^ CCR7^–^ effector memory) CD4^+^ and CD8^+^ T cells were sorted from PBMCs and expanded polyclonally in multiple cultures to generate T cell libraries of 48–192 cell lines [13]. Each cell line was then screened for reactivity with peptide pools spanning each of the four HCMV proteins IE-1, pp65, the pentamer gHgLpUL128L and gB. Responding cell lines were detected by [3H]-thymidine incorporation and frequency was calculated using the Poisson distribution [13].

Naïve CD4^+^ T cells reactive with one or more HCMV proteins were detected in four out of six HCMV seronegative donors at frequencies ranging from <2 to 21 per 10^6^ cells (Fig 1A and 1B). Naïve CD8^+^ T cells were detected in all six donors at frequencies ranging from <2 to 124 per 10^6^ cells. In both cases, the pentamer gHgLpUL128L appeared to be the immunodominant target of naïve T cells, although after normalization of the dataset for protein size, the estimated frequencies were comparable for the four proteins tested (S1A and S1B Fig).

As expected, a high frequency of HCMV-reactive cells was detected in the memory CD4^+^ and CD8^+^ compartment at the early time point after primary HCMV infection. However, in contrast to that observed with naïve T cells, the envelope protein gB and the abundant tegument protein pp65 were the immunodominant targets of memory CD4^+^ T cells, while the non-structural IE-1 protein and pp65 were the immunodominant targets of CD8^+^ T cells (Fig 1C and 1D, and S1C and S1D Fig). This pattern of reactivity was maintained in the late stages of infection, although frequencies of HCMV-specific CD4^+^ and CD8^+^ T cells (particularly IE-1- and pp65-specific) decreased (Fig 1C and 1D, and S1C and S1D Fig).

In donors with remote infection, the distribution of protein-specific T-cell frequencies grossly followed the same trend as in donors with primary infection, although frequencies of HCMV-specific CD8^+^ T cells were markedly lower (Fig 1E and 1F, and S1E and S1F Fig).

Taken together, these data suggest that different frequencies of protein specific T cells are elicited by HCMV infection and are not predetermined in the naïve compartment, but are the result of virus-host interactions.

### T-cell specificity for HCMV proteins and virus transmission to the fetus

**Fig 2:**
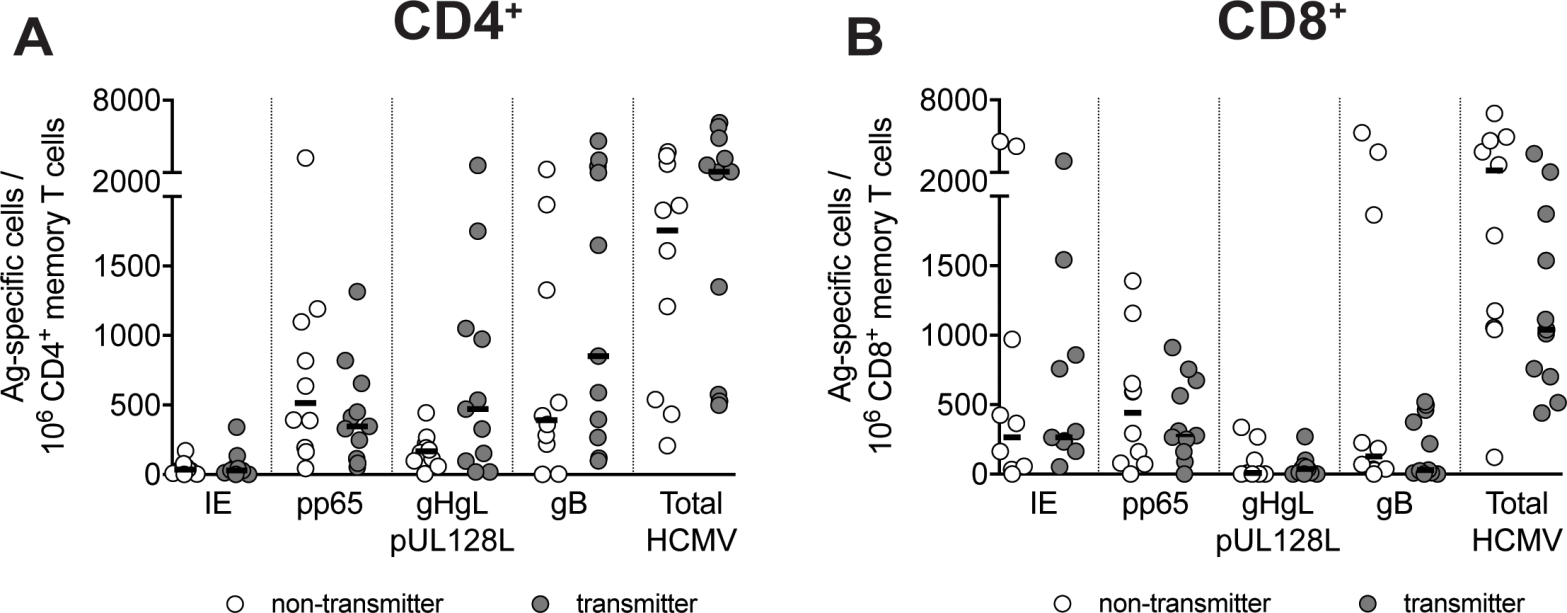
Pregnant women transmitting or non-transmitting HCMV to the fetus have comparable frequencies of specific T cells at early time points after infection. Frequencies of protein-specific **(A)** CD4^+^ and **(B)** CD8^+^ memory T cells in transmitting (n = 11) or non-transmitting mothers (n = 10) tested one month after HCMV infection onset are given. The total of HCMV-specific T cells is the sum of the single protein-specific T cells. Each symbol represents an individual and horizontal black lines indicate median values. No significant P value, as determined by Mann-Whitney U test, was found between T and NT women.

Subsequently, we studied 21 pregnant women developing HCMV infection within the first or second trimester of pregnancy; 11 of them transmitted (T) and 10 did not transmit (NT) the virus to the fetus. The pattern of antigen specificity in CD4^+^ and CD8^+^ T cells was similar to that described for non-pregnant patients (Fig 1C and 1D). The individual CD4^+^ or CD8^+^ T cell frequencies specific for each of the four proteins, as well as the sum of frequencies for all four HCMV proteins were not significantly different between T or NT women (Fig 2). This indicates that the T-cell specific reactivity against any of the four proteins examined was not associated with protection from HCMV transmission to the fetus.

### Distribution of HCMV-specific CD4^+^ and CD8^+^ T cells among short-term effector (IL-7R^neg^) and long-term memory (IL-7R^pos^) cells at different stages of infection

**Fig 3:**
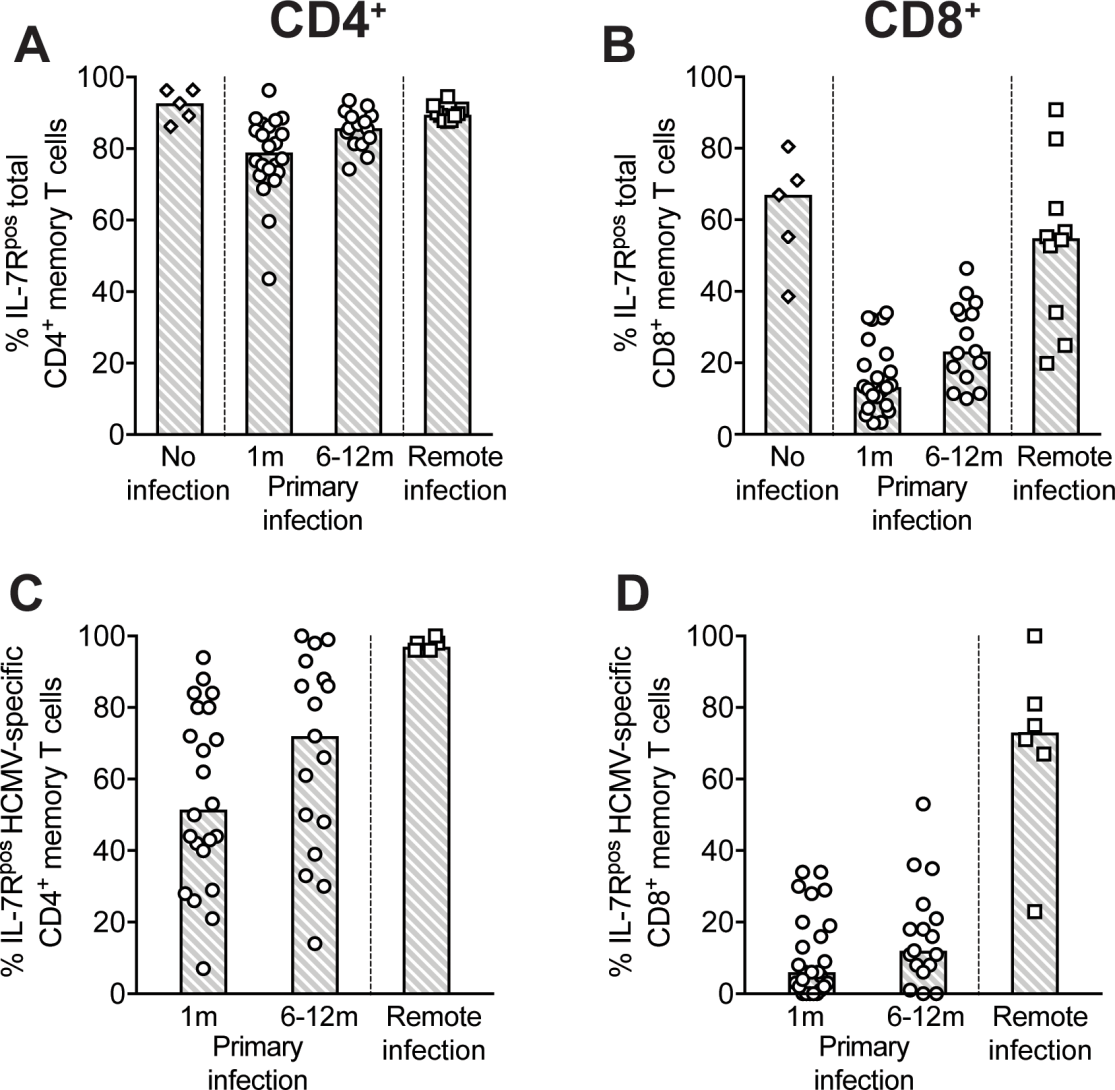
IL-7R^neg^ short-term effector _vs_ IL-7R^pos^ long-term memory T cells persistance at late time points after primary HCMV infection. Frequencies of IL-7R^pos^ T cells in **(A-B)** total memory and **(C-D)** HCMV-specific CD4^+^ or CD8^+^ T cells are reported. Data are from HCMV-seronegative subjects (“No infection”, n = 5, only for total memory), patients (both pregnant and non-pregnant) within 1 month (n = 25) or at 6–12 months (n = 18) after primary infection onset, and subjects with remote infection (n = 10). “HCMV-specific” indicates the sum of the single protein-specific T cells. Each symbol represents an individual and column upper limits indicate median values.

It is known that following antigenic stimulation, naive T cells proliferate and differentiate into IL-7R^neg^ effector T cells and IL-7R^pos^ memory T cell precursors [15–17]. We therefore analyzed the frequency of IL-7R^neg^ and IL-7R^pos^ T-cell subsets in the total CD4^+^ and CD8^+^ compartments (see representative staining in S2 Fig). In acute HCMV infection, the frequency of IL-7R^pos^ cells in the total CD4^+^ T-cell memory compartment slightly decreased with respect to HCMV-naïve donors and then increased (Fig 3A) towards the basal level (remote infections). However, 6–12 months after infection, total IL-7R^pos^ CD4^+^ T cells still did not reach the basal level. This phenomenon was much more evident in the memory CD8^+^ T-cell compartment, where only 10–20% of T cells were IL-7R^pos^ (while the great majority were IL-7R^neg^) one month after infection onset (Fig 3B), thereafter increasing slowly towards basal levels (remote infections).

The phenotype of HCMV-specific T cells according to IL-7R expression was then analyzed. Since T cells specific for each HCMV protein followed the same pattern, the sum of protein-specific T cells was considered a surrogate of HCMV-specificity. About 50% of HCMV-reactive CD4^+^ T cells (median value) were IL-7R^pos^ one month after infection onset, and then increased until comprising virtually all of the circulating HCMV-specific CD4^+^ T cells in remote infections (Fig 3C). As expected, T cells specific for other previously encountered viral antigens (Flu and RSV) were detected only among the IL-7R^pos^ memory subset and not in the IL-7R^neg^ subset (data not shown). Interestingly, only 10–20% of HCMV-specific CD8^+^ T cells were IL-7R^pos^ long-term memory cells up to 12 months after primary infection, subsequently increasing to 70% in remote infection (Fig 3D). However, there was a great variation in values among CD4^+^ T cells, indicating that some subjects develop HCMV-specific CD4^+^ T cells with an IL-7R^pos^ phenotype early after infection, whereas other subjects maintain a high frequency of IL-7R^neg^ HCMV-specific CD4^+^ T cells for long periods of time (Fig 3C).

In the CD8^+^ subset, the circulating HCMV-specific T cell pool was dominated by the IL-7R^neg^ population and IL-7R^pos^ long-term memory cells reached a substantial level one year or later after infection.

### IL-7R^neg^ *vs* IL-7R^pos^ HCMV-specific T cells after primary infection and virus transmission to the fetus

**Fig 4:**
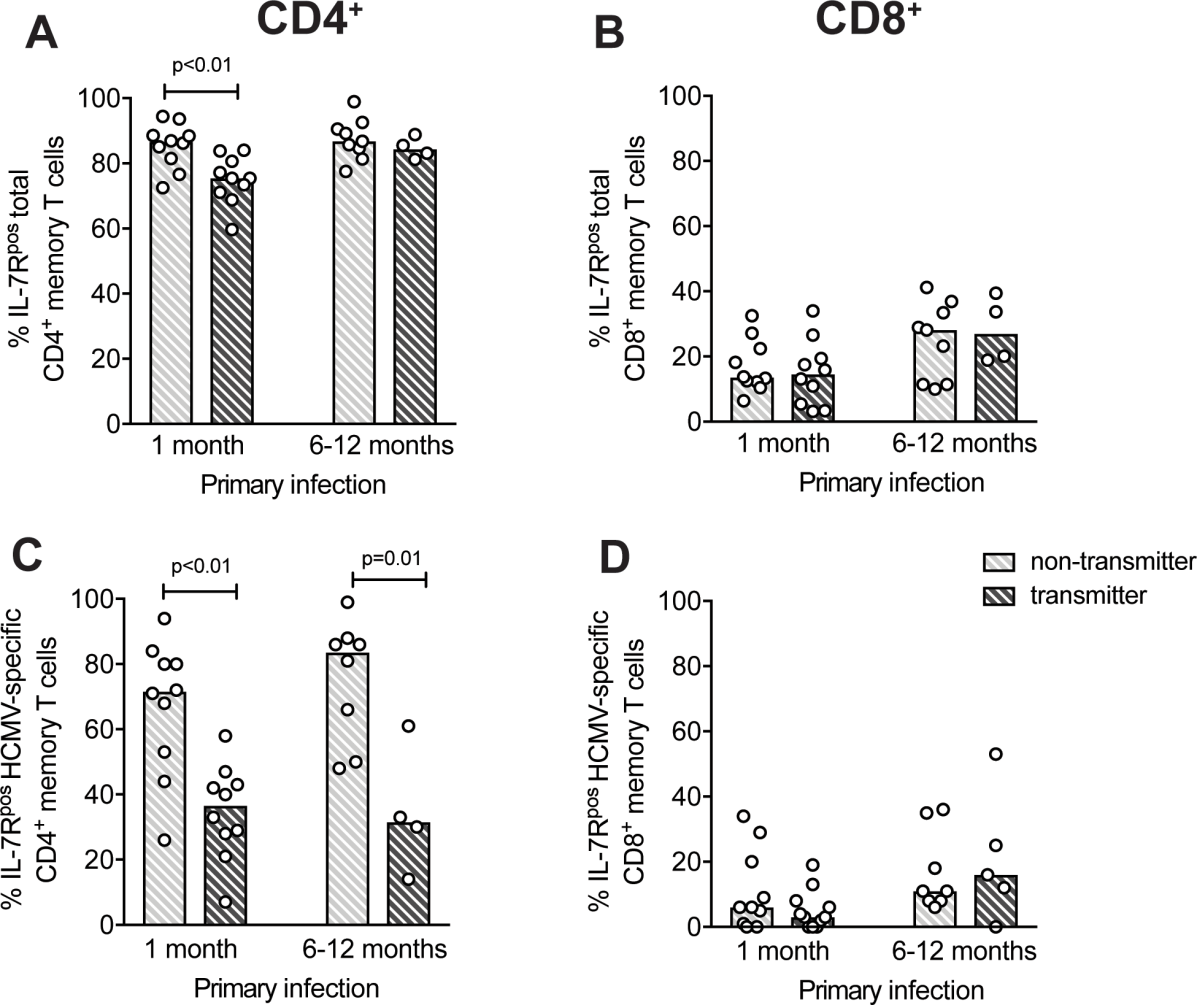
Pregnant women non-transmitting HCMV to the fetus have higher frequencies of long-term IL-7R^pos^ memory CD4^+^ T cells than transmitting women. Percentages of IL-7R^pos^ T cells among **(A, B)** total memory or **(C, D)** HCMV-specific CD4^+^ or CD8^+^ T cells in pregnant women transmitting or non-transmitting the infection to the fetus (n = 11 and n = 10, respectively) analyzed 1 month or 6–12 months (n = 8 and n = 4, respectively) after primary infection are reported. “HCMV-specific” indicates the sum of the single protein-specific T cells. Each symbol represents an individual and column upper limits indicate median values. P values were determined by Mann-Whitney U test.

In order to verify whether the HCMV-specific T-cell phenotype is associated with the risk of virus transmission to the fetus and because of the variable proportion of IL-7R^pos^ *vs* IL-7R^neg^ T cells in different subjects, the percentage of HCMV-specific T cells with the IL-7R^pos^ subset was compared between T and NT pregnant women. When we looked at total memory CD4^+^ and CD8^+^ T cells (Fig 4A and 4B), it was found that total CD4^+^ T cells of NT women showed a significantly higher percentage of IL-7R^pos^ cells, at only one month after infection onset (Fig 4A). Interestingly, on the other hand, NT women showed a significantly higher frequency of HCMV-specific CD4^+^ T cells with a IL-7R^pos^ phenotype than T women, both 1 month and 6–12 months after infection (Fig 4C). Instead, no difference was observed for HCMV-specific CD8^+^ T cells (Fig 4D). Collectively, these data suggest that the early appearance of CD4^+^ T cells with an IL-7R^pos^ long-term memory phenotype is associated with a lower risk of HCMV transmission to the fetus.

### Characterization of IL-7R^neg^ and IL-7R^pos^ CD4^+^ and CD8^+^ T cells in the context of HCMV infection

The activation status and cytotoxic potential of circulating IL-7R^neg^ and IL-7R^pos^ CD4^+^ and CD8^+^ memory T cells was investigated by directly analyzing *ex vivo* the expression of Ki67, HLA-DR, and PD-1 as activation markers, and perforin as a marker of cytotoxicity (see S3 Fig for a representative experiment).

**Fig 5:**
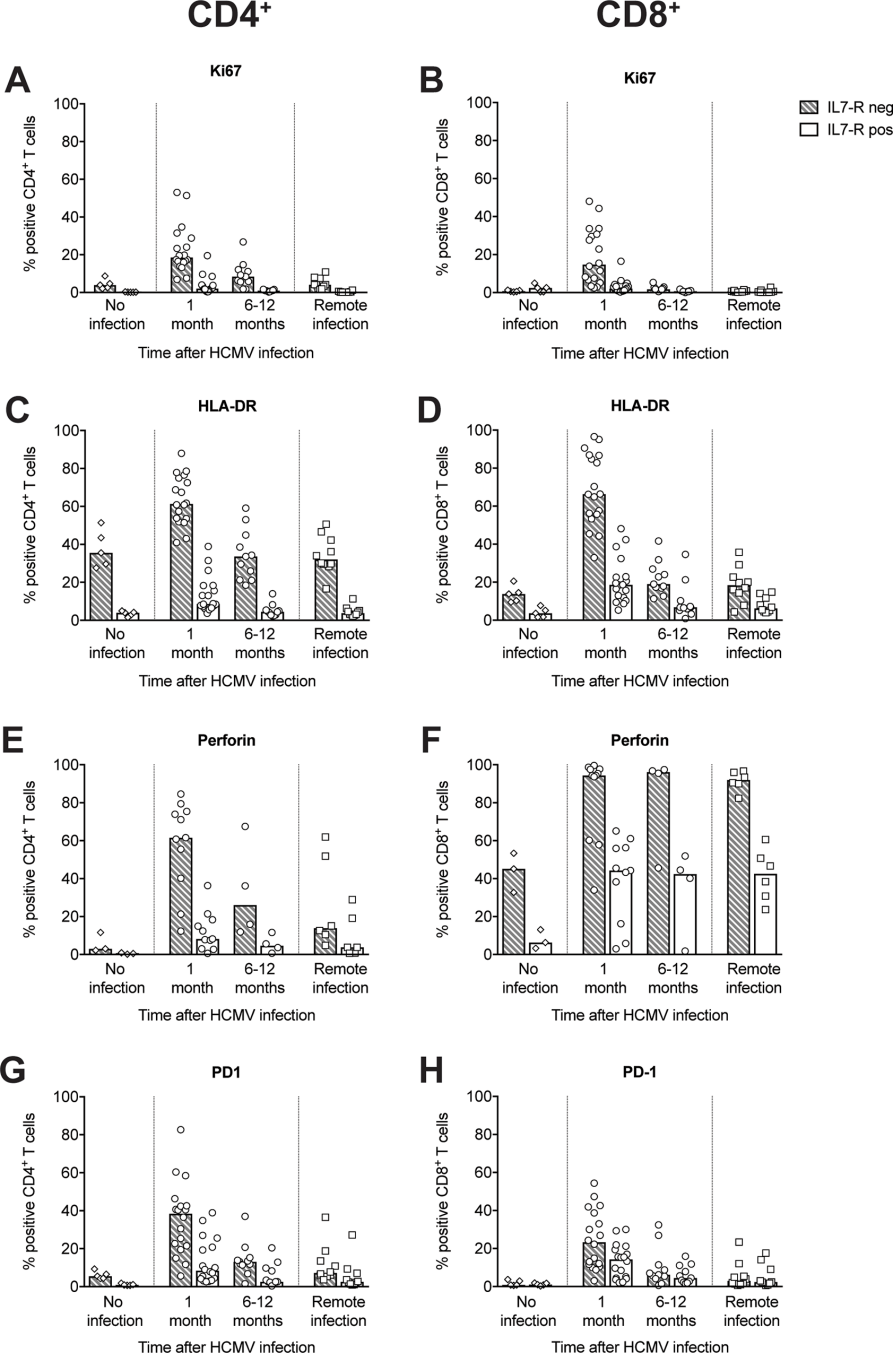
Characterization of IL-7R^neg^ and IL-7R^pos^ subsets among total memory T cells in subjects at different stages of HCMV infection. Data are reported for HCMV-seronegative subjects (“No infection”, n = 5), patients with primary HCMV infection 1 (n = 18) or 6–12 months (n = 11) after infection onset, or subjects with remote HCMV infection (n = 10). Percentages of CD4^+^ (left panels) and CD8^+^ (right panels) T cells in IL-7R^neg^ and IL-7R^pos^ subsets expressing **(A,B)** Ki67, **(C,D)** (HLA-DR, **(E,F)** perforin, and **(G,H)** PD-1 are shown. Each symbol represents an individual, and column upper limits indicate median values.

In general, IL-7R^neg^ CD4^+^ and CD8^+^ T cells exhibited a much more activated phenotype than IL-7R^pos^ cells one month after infection, as shown by the elevated expression of Ki67, indicating recent proliferation, and HLA-DR, indicating cell activation (Fig 5A–5D). The activation status subsequently decreased, reaching a steady state 6–12 months after infection. IL-7R^neg^ CD4^+^ and CD8^+^ T cells also exhibited a more cytotoxic phenotype than their IL-7R^pos^ counterparts (Fig 5E and 5F). Almost all IL-7R^neg^ CD8^+^ T cells expressed perforin both in acute and remote infection. In contrast, perforin expression in IL-7R^neg^ CD4^+^ T cells was high in the first month after infection (60% of cells produced perforin); this perforin expression subsequently decreased. Perforin expression remained higher in CD4^+^ and CD8^+^ T cells of subjects with remote infection compared to expression in seronegative subjects.

One month after infection, 38% of CD4^+^ and 23% of CD8^+^ IL-7^neg^ T cells expressed PD-1, a marker of T-cell activation/exhaustion (Fig 5G and 5H). Consistent with an exhausted phenotype, IL-7R^neg^ T cells had a lower mean cloning efficiency (CD4^+^: 31%; CD8^+^: 38%) than IL-7R^pos^ T cells (CD4^+^: 78%; CD8^+^: 76%). The low cloning efficiency of IL-7R^neg^ T cells was still observed 6–12 months after infection (data not reported).

Collectively these results show that IL-7R^neg^ T cells are highly activated only in the early stage, although they persist for a long period after infection. In contrast, the high cytotoxic potential of IL-7R^neg^ CD8^+^ T cells does not change with time.

## Discussion

In the present study, we used a high-throughput cell screening assay to investigate the pre-immune T cell repertoire in HCMV seronegative subjects and the development of the T-cell response to HCMV in the immunocompetent host. In particular, we analyzed the relationship between the T-cell response in pregnancy and congenital HCMV infection. In detail, we evaluated the T cell phenotype: short-term effector *vs* long-term memory cells (according to IL-7R expression) and its correlation with protection from virus transmission to the fetus.

Our analysis, which focused on the HCMV proteins IE-1, pp65, the pentamer gHgLpUL128L, and gB (all are widely investigated in vaccine and immune monitoring studies), indicates a similar naïve T-cell frequency among different specificities, suggesting that the compartmentalization of T-cell specificity is not pre-determined in the naïve T-cell repertoire. However, after infection, effector/memory CD4^+^ T cells specific for gB and pp65 and effector/memory CD8^+^ T cells specific for IE-1 and pp65 become immunodominant. The variable frequency of T cells specific for the viral proteins in the pre- and post-exposure repertoire may be due to different levels of protein synthesis and antigen availability. It is likely that non-structural IE-1 protein, although abundantly synthesized in infected cells, is poorly available in the extracellular compartment for phagocytosis and presentation on MHC-II molecules to CD4^+^ T cells. On the contrary, the envelope glycoprotein complexes present on the surface of virions and dense bodies are less abundantly produced, but have better access to the extracellular compartment. Among envelope glycoproteins, gB, which is more abundant than the pentamer components pU128, pUL130 and pUL131 [18], induces a higher frequency of specific T cells. Finally, pp65 is the most abundantly synthesized protein having access to both the extracellular and cytoplasmic compartment, even before virus replication [19]. Thus, it can efficiently stimulate both CD4^+^ and CD8^+^ T cells.

The finding that the four HCMV proteins analyzed share the same potential for recognition by naïve T cells has important implications for vaccine formulation. We and others have described the gHgLpUL128L pentamer complex as the most potent in eliciting the most efficient neutralizing antibody response after natural infection of humans and animal immunization [20–22]. We found than the pentamer also elicits a CD4^+^ T helper-cell response *in vivo*, although to a lower frequency with respect to gB and pp65. However, since the frequency of naïve T cells specific for the pentamer is similar to that of the other proteins examined, it is possible that a subunit vaccine based on the complex alone could also induce an efficient T-cell response, when administered at an optimal concentration and in an appropriate formulation.

The analysis of the T cell specificity elicited in pregnant women with primary HCMV infection showed no association between the frequency of T cells specific for any of the four HCMV proteins examined and virus transmission to the fetus. Thus, T-cell specificity does not appear to be preferentially indicative of protection in this clinical setting. However, we cannot exclude that other proteins are involved in eliciting a protective T-cell response, although they have been less widely investigated [14].

Interesting data on protection from HCMV infection arose from the analysis of the T cell response in the two cellular compartments identified according to IL-7R expression [15,16]. IL-7R signaling is known to be required for the homeostatic maintenance of naïve and memory T cells [23] and human naïve T cells rapidly downregulate IL-7R expression upon antigen stimulation via TCR/CD28 [24]. In murine models, it has been suggested that a small subset of activated T cells expressing IL-7R during primary infection give rise to long-term memory cells, while IL-7R^neg^ short-term effectors are destined to die after antigen clearance [15,16].

It is not known whether these memory precursors maintain or re-acquire IL-7R expression after initial activation. However, in the case of HCMV infection in humans, we found that IL-7R^neg^ short-term effectors persist for a long time after primary infection, especially in the CD8^+^ compartment: one year after infection the majority (ca. 80%) of CD8^+^ and a proportion of CD4^+^ HCMV-specific T cells do not express IL-7R. At later time points, survival of HCMV-specific IL-7R^neg^ cells appears to be a property of CD8^+^ and not CD4^+^ T cells. The open question is whether IL-7R^neg^ cells generated during primary infections are subsequently maintained by homeostatic stimuli other than IL-7, such as IL-15 [24, 25], or by continuous contact with antigen due to HCMV persistence and reactivation [17, 26]. In the latter case, they would not really be short-term effectors. Alternatively, they could be very short-lived cells continuously generated from naïve or memory cells in response to HCMV reactivations. However, we found that IL-7R^neg^ cells are highly activated in the early, but not in the late stages of infection, suggesting that they survive in the resting memory pool after initial activation during the primary response, rather than being continuously generated.

Strikingly, when T and NT pregnant women were examined separately, a significantly lower percentage of IL-7R^neg^ (and, thus, higher percentage of IL-7R^pos^) HCMV-specific CD4^+^ T cells was associated with a reduced risk of virus transmission to the fetus. The higher percentage of IL-7R^pos^ HCMV-specific CD4^+^ T cells in NT women was observed also at 6–12 months after infection, although a smaller number of women were examined at this late time point. We can speculate that mothers with a higher cumulative exposure to virus and viral antigens are at greater risk of transmitting the virus to the fetus. In parallel, the higher exposure to HCMV would also be responsible for the lower frequency of IL-7R^pos^ and higher frequency of IL-7R^neg^ HCMV-specific CD4^+^ T cells observed in T mothers. Alternatively, T may differ from NT mothers in immune responsiveness to HCMV. Thus, it is possible that a higher percentage of IL-7R^pos^ CD4^+^ T cells is the result of better virus control, either by first line innate immune defenses or by a rapid generation of the adaptive immune system. In turn, this would lead to rapid virus clearance and a lower risk of virus transmission to the fetus. These hypotheses cannot be experimentally addressed in humans, but are both plausible and in agreement with findings reported by other investigators on the relationship between antigen exposure and frequency of IL-7R^pos^ T cells. In line with these hypotheses, it was found that in kidney transplant recipients, the percentage of IL-7R^neg^ HCMV specific CD8^+^ T cells correlates with peak viral load in the acute phase of infection [17], while a higher concentration of inflammatory cytokines during T-cell priming promotes IL-7R^neg^ *vs* IL-7R^pos^ CD8^+^ T cells [27], and strong T-cell stimulation during priming determines a low frequency of IL-7R^pos^ CD4^+^ T cells [28].

In this study, we did not detect a significantly lower viral load in blood from NT women, as reported in other studies [12, 29]. However, it should be kept in mind that HCMV load in blood of immunocompetent subjects with primary infection is usually low and close to the detection limit of available assays [30]. Moreover, frequent sequential monitoring of HCMV DNA after infection was not feasible.

The sustained presence of IL-7R^neg^ HCMV-specific T cells could also explain the long time required to detect a lymphoproliferative response to HCMV after primary infection [7, 8]: IL-7R^neg^ cells are more exhausted (higher PD-1 expression) and have a lower proliferative potential (lower cloning efficiency). Thus, in the early phase after infection, when IL-7R^neg^ short -term effectors are predominant in the HCMV-specific T-cell pool, the proliferation of PBMC in response to HCMV antigen stimulation *in vitro* is poor. When the IL-7R^pos^ counterpart increases (and, conversely, IL-7R^neg^ decreases), the lymphoproliferative response to HCMV would also increase in parallel. Accordingly, the lower lymphoproliferative response observed in T *vs* NT women [7, 8] is associated with the higher percentage of IL-7R^neg^ HCMV-specific CD4^+^ T cells observed in T women.

Interestingly, and in agreement with previous observations obtained in humans and in macaques [7, 8, 31], we confirm that the maternal CD4^+^ T cell response plays a pivotal role in the prevention of HCMV congenital infection. Moreover, the fact that IL-7R^neg^ cells have greater cytotoxic potential, but are less important for protection may indicate that cytotoxicity is not the main T-cell function associated with HCMV control. This is in line with the more important role in protection that appears to be associated with CD4^+^ T cells.

In conclusion, the specific antigen reactivity of T cells does not seem to correlate with protection from virus transmission to the fetus. On the other hand, the rapid appearance of HCMV-specific CD4^+^ T cells with a long-term memory *vs* short-term effector phenotype appears to be associated with a lower risk of HCMV transmission to the fetus. This finding indicates that the analysis of the HCMV-specific T-cell immune response phenotype may help to define reliable correlates of maternal immune protection from vertical virus transmission, and IL-7R expression may be a predictive marker of protection.

## Methods

### Study subjects

Blood samples were obtained from 27 immunocompetent adult patients undergoing primary HCMV infection: 21 pregnant women, undergoing primary HCMV infection within the first or second trimester of pregnancy, and 6 subjects (3 males, 3 females) with symptomatic primary HCMV infection, referred to the Fondazione IRCCS Policlinico San Matteo, Pavia, Italy. In addition, 6 HCMV seronegative and 10 HCMV-seropositive healthy adult subjects (with remote HCMV infection occurring >5 years prior) were enrolled. All subjects were Caucasian. Diagnosis and timing of primary HCMV infection was based on two or more of the following: IgG seroconversion, kinetics of HCMV-specific IgM antibody, IgG avidity index, detection of HCMV DNA in blood [32, 33]. Appearance of symptoms and biochemical/hematological signs as well as presence of HCMV DNA in blood and/or antibody kinetics were used to determine onset of infection [34]. Congenital infection was diagnosed by detection of HCMV DNA in newborn urine samples collected within the first two weeks of life.

### Ethics statement

Samples (all codified and anonymized) were collected for a study of the immune response to HCMV during primary infection and its protective role against virus transmission to fetus. The study was approved by the Institutional Review Board and Bioethics Committee of the Fondazione IRCCS Policlinico San Matteo and all participants gave their written informed consent. Mothers gave written informed consent for diagnostic testing of urine samples from their newborns.

### Sorting of naïve and memory T-cell subsets

Cryo-preserved PBMC from an already existing collection were thawed and, after resting for 2-3h in complete culture medium (see below) at 37°C, they were stained with the following monoclonal antibodies before sorting of T-cell subsets with a FACSAria instrument (BD Biosciences, San Jose, CA, USA): anti-CD4-PE-Texas Red (Invitrogen, Frederick, MD, USA), anti-CD8-FITC (Beckman Coulter-Immunotech, Marseille, France), anti-CD45RA-Qdot655, (Life Technologies, Eugene, OR, USA), anti-CCR7-BV421 (BioLegend Inc., San Diego, CA, USA), anti-CD127 (IL-7R)-PE (BD Biosciences). CD45RA^+^ CCR7^+^ naïve T cells were sorted from HCMV-seronegative subjects, whereas total memory T cells were sorted from HCMV-seropositive subjects and further divided into IL-7R^pos^ and IL-7R^neg^ subsets.

### Determination of protein-specific T-cell frequency by the library method

After sorting, naïve and memory CD4^+^ and CD8^+^ T cells were divided into numerous replicate cultures (48 replicates of 250–500 cells for memory and 96–192 replicates of 2000 cells for naïve T cells) and expanded for three weeks with 1 μg/mL Phytohemoagglutinin-L (PHA) in the presence of irradiated (45 Gy) allogeneic feeder cells (10^5^ cells per well) and IL-2 (500 IU/ml) in a 96-well plate format, as previously described [13]. Then, aliquots from each culture were tested in parallel for their capacity to proliferate in response to 2 μg/ml peptides (15-mer, overlapping by 10 aminoacids) spanning the following entire proteins or protein complexes: IE-1, pp65, the pentamer gHgLpUL128L, gB (all from A&A Labs LLC, San Diego, CA). In some experiments, peptides from influenza virus (Flu: Matrix protein 1, Nucleoprotein and Neuraminidase) and respiratory syncytial virus (RSV: Fusion protein and Nucleoprotein) were used as control antigens. Proliferation was measured after two days of culture, with the last 16-h incubation in the presence of 1 μCi/ml [methyl-3H]-thymidine (Perkin-Elmer, Waltham, MA, USA). The frequency of specific precursors was calculated according to Poisson’s distribution. Since memory T cells were sorted into IL-7R^pos^ and IL-7R^neg^ subsets, the frequency of total memory HCMV-specific T cells was calculated as the sum of the frequencies of IL-7R^pos^ and IL-7R^neg^ HCMV-specific T cells.

### Cloning efficiency of T-cell subsets

IL-7R^pos^ and IL-7R^neg^ T cells were plated in 384 well plates at a concentration of 0.6 cells/well and expanded with 1 μg/mL PHA in the presence of irradiated (45 Gy) allogeneic feeder cells (10^5^ cells per well) and IL-2 (500 IU/ml). After 7–10 days culture, the number of wells containing expanded T cell clones were counted and the cloning efficiency was calculated according to Poisson’s distribution as the percentage of plated cells giving rise to proliferating clones.

### Flow cytometry analysis of circulating memory T-cell subsets

After resting 2-3h in complete medium at 37°C, thawed PBMC were stained with the following mAbs: anti-CD4-Alexa Fluor 700 (BioLegend Inc., San Diego, CA, USA), anti-CD8-PE-Texas Red (Invitrogen), anti-CD45RA-Qdot655, anti-CCR7-BV421, anti-HLA-DR-V500 (BD Biosciences), and anti-PD-1-BV711 (BioLegend). Cells were subsequently fixed and permeabilized with Cytofix/Cytoperm (BD Biosciences), stained with anti-Ki67-PerCP eFluor710 (eBioscences, San Diego, CA) and anti-perforin-FITC (Diaclone, Besançon, France), and acquired with a LSR II Fortessa flow cytometer (BD Biosciences). Analysis was performed with FlowJo software (FlowJo LLC, Ashland, OR); after gating on total memory CD4^+^ and CD8^+^ lymphocytes, the percentage of IL-7R^pos^ and IL-7R^neg^ cells was calculated, as well as the percentage of IL-7R^pos^ and IL-7R^neg^ CD4^+^ T cells expressing Ki67, HLA-DR, PD-1 and perforin.

### Statistical analysis

The Mann-Whitney U test was used to compare frequencies of protein-specific T cells and the percentage of IL-7R^neg^ HCMV-specific T cells between T and NT women.

## Supporting information

S1 Fig Normalized frequencies of HCMV-specific CD4^+^ and CD8^+^ T cells in seronegative subjects and in subjects with primary or remote HCMV infection. Frequencies of T cells specific for the HCMV proteins IE-1 (491 aa), pp65 (561 aa), gHgLpUL128L (1535 aa, the pentamer) and gB (905 aa) shown in Fig 1 were normalized according to protein length, using IE as a reference. Normalized frequencies are reported for HCMV-specific CD4^+^ and CD8^+^ naïve T cells in (**A,B**) 6 HCMV- seronegative subjects, and HCMV-specific CD4^+^ and CD8^+^ memory T cells in (**C,D**) 6 patients with primary HCMV infection tested one month (grey symbols) and 6–12 months (white symbols) after infection onset, and in (**E,F**) 7 subjects with remote HCMV infection. Each symbol represents an individual, and horizontal black lines indicate median values.
(TIF)

S2 Fig Sorting strategy for IL-7R positive and negative memory T cell subsets. After gating on total memory T cells according to the expression of CD45RA and CCR7 (i.e. after exclusion of CD45RA^+^/CCR7^+^ CD4^+^ or CD8^+^ T cells), lymphocytes were divided according to their expression of IL-7R. Plots are from a representative patient analyzed (**A**) one and (**B**) 12 months after infection onset.
(PPTX)

S3 Fig Characterization of IL-7R^pos^ and IL-7R^neg^ T cells in a representative patient at 1 and 12 months after onset of primary HCMV infection. Expression of (**A,B**) Ki-67, (**C,D**) HLA-DR, (**E,F**) perforin, and (**G,H**) PD-1 *vs* IL-7R in gated total memory CD4^+^ and CD8^+^ T cells.
(PPTX)
