# Supplementary figures and images for: Phenotype and specificity of T cells in primary human cytomegalovirus infection during pregnancy: IL-7Rpos long-term memory phenotype is associated with protection from vertical transmission
Source: PLoS One. 2017 Nov 7;12(11):e0187731. doi: 10.1371/journal.pone.0187731 (PMC5675411; doi:10.1371/journal.pone.0187731)

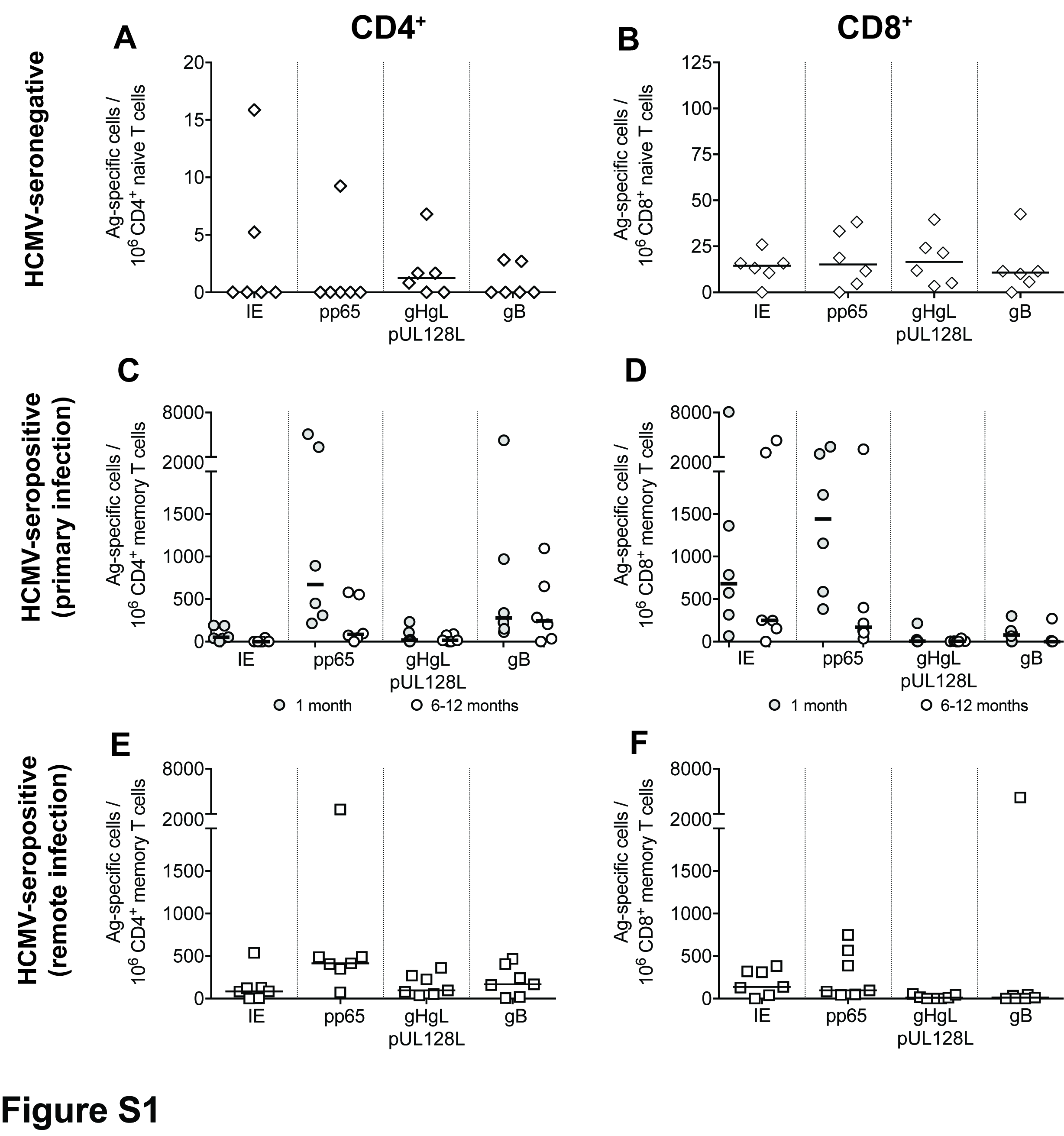

Supplement: S1 Fig — Frequencies of T cells specific for the HCMV proteins IE-1 (491 aa), pp65 (561 aa), gHgLpUL128L (1535 aa, the pentamer) and gB (905 aa) shown in Fig 1 were normalized according to protein length, using IE as a reference. Normalized frequencies are reported for HCMV-specific CD4+ and CD8+ naïve T cells in (A,B) 6 HCMV- seronegative subjects, and HCMV-specific CD4+ and CD8+ memory T cells in (C,D) 6 patients with primary HCMV infection tested one month (grey symbols) and 6–12 months (white symbols) after infection onset, and in (E,F) 7 subjects with remote HCMV infection. Each symbol represents an individual, and horizontal black lines indicate median values. (TIF) [file pone.0187731.s001.tif]
