# Supplementary material for: Phenotype and specificity of T cells in primary human cytomegalovirus infection during pregnancy: IL-7Rpos long-term memory phenotype is associated with protection from vertical transmission
Source: PLoS One. 2017 Nov 7;12(11):e0187731. doi: 10.1371/journal.pone.0187731 (PMC5675411; doi:10.1371/journal.pone.0187731)
Supplement: S2 Fig — After gating on total memory T cells according to the expression of CD45RA and CCR7 (i.e. after exclusion of CD45RA+/CCR7+ CD4+ or CD8+ T cells), lymphocytes were divided according to their expression of IL-7R. Plots are from a representative patient analyzed (A) one and (B) 12 months after infection onset. (PPTX) [file pone.0187731.s002.pptx]

## Slide 1
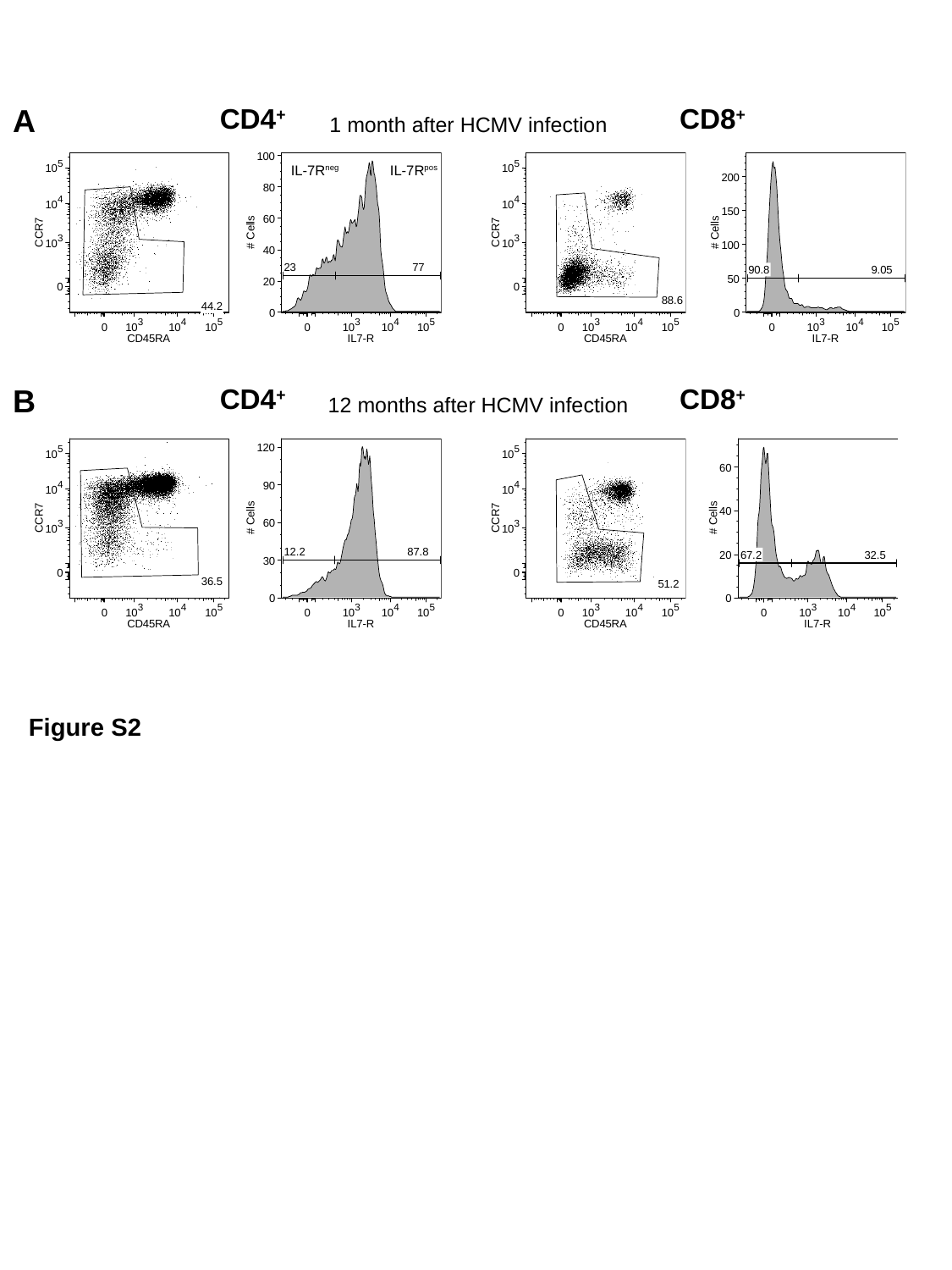

A
CD4+
CD8+
1 month after HCMV infection
IL-7Rneg
IL-7Rpos
B
CD4+
CD8+
12 months after HCMV infection
Figure S2
