# Supplementary material for: Phenotype and specificity of T cells in primary human cytomegalovirus infection during pregnancy: IL-7Rpos long-term memory phenotype is associated with protection from vertical transmission
Source: PLoS One. 2017 Nov 7;12(11):e0187731. doi: 10.1371/journal.pone.0187731 (PMC5675411; doi:10.1371/journal.pone.0187731)
Supplement: S3 Fig — Expression of (A,B) Ki-67, (C,D) HLA-DR, (E,F) perforin, and (G,H) PD-1 vs IL-7R in gated total memory CD4+ and CD8+ T cells. (PPTX) [file pone.0187731.s003.pptx]

## Slide 1
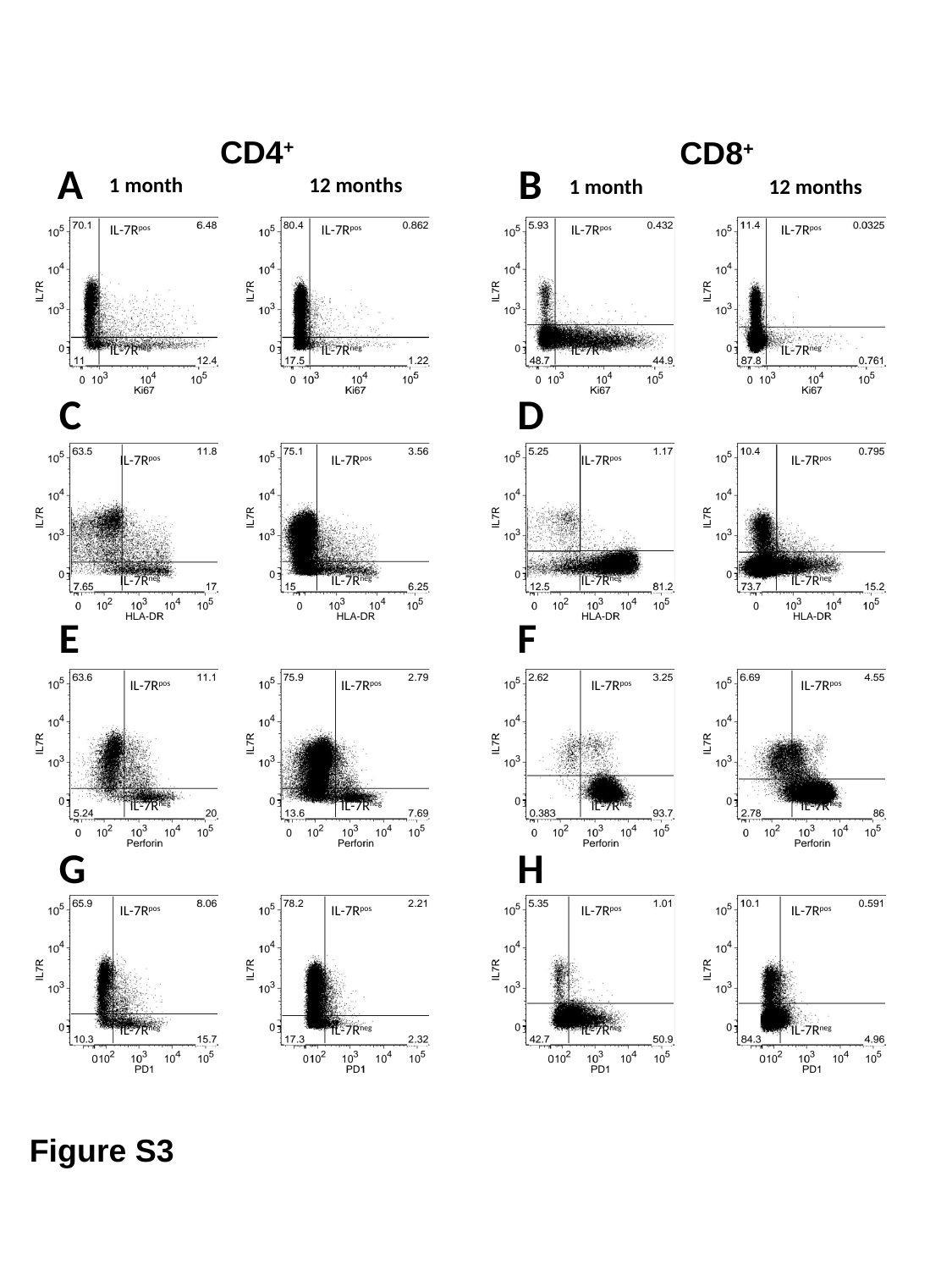

CD4+
CD8+
A
B
1 month
12 months
1 month
12 months
IL-7Rpos
IL-7Rpos
IL-7Rpos
IL-7Rpos
IL-7Rneg
IL-7Rneg
IL-7Rneg
IL-7Rneg
C
D
IL-7Rpos
IL-7Rpos
IL-7Rpos
IL-7Rpos
IL-7Rneg
IL-7Rneg
IL-7Rneg
IL-7Rneg
E
F
IL-7Rpos
IL-7Rpos
IL-7Rpos
IL-7Rpos
IL-7Rneg
IL-7Rneg
IL-7Rneg
IL-7Rneg
G
H
IL-7Rpos
IL-7Rpos
IL-7Rpos
IL-7Rpos
IL-7Rneg
IL-7Rneg
IL-7Rneg
IL-7Rneg
Figure S3
